# Supplementary material for: Content-rich biological network constructed by mining PubMed abstracts
Source: BMC Bioinformatics. 2004 Oct 8;5:147. doi: 10.1186/1471-2105-5-147 (PMC528731; doi:10.1186/1471-2105-5-147)
Supplement: Additional File 5 — The original Chilibot query results of the term "long-term potentiation (LTP)" and 22 other terms, limiting the latest references analyzed to the years 1990, 1995, 2000, and 2004. [file 1471-2105-5-147-S5.bz2 › chilibotAdditionalFile5/ltp2000/html/LTP_ACTIN.html]

 


 **LTP** and **ACTIN** 
  
Found 17 abstracts in PubMed,  **17 abstracts were retrieved and analyzed**.  


---

 Search Google  |
 PDF files only 
|  EDU domain only 

---

**Interactive relationship** (e.g. stimulation, inhibition, etc)

- These results suggest that postsynaptic  **actin**  filaments are involved in a dynamic process required to maintain AMPAR mediated transmission and to enhance it during  **LTP** .  Ref: 10341235 J Neurosci, 1999
- Dynamic  **actin**  filaments are required for stable long term potentiation  [ **LTP** ]   **LTP**  in area CA1 of the hippocampus.  Ref: 10823894 Proc Natl Acad Sci U S A, 2000
- The results are consistent with a model in which dynamic  **actin**  filaments play an essential role in the molecular mechanisms underlying the early maintenance phase of  **LTP** , such as growth of new synaptic connections or conversion of silent synapses.  Ref: 10823894 Proc Natl Acad Sci U S A, 2000

**Parallel relationship** (e.g. studied together, co-existance, homology, etc.)

- **Actin**  in synaptic cytoskeleton during long term potentiation  [ **LTP** ]  in hippocampal slices.  Ref: 1811261 Acta Histochem Suppl, 1991
- A role of  **actin**  filament in synaptic transmission and long term potentiation  [ **LTP** ] .  Ref: 10341235 J Neurosci, 1999
- **Actin**  study of the synapses of surviving hippocampal slices during long term potentiation  [ **LTP** ]  .  Ref: 3413824 Tsitologiia, 1988
